# Supplementary material for: Content-rich biological network constructed by mining PubMed abstracts
Source: BMC Bioinformatics. 2004 Oct 8;5:147. doi: 10.1186/1471-2105-5-147 (PMC528731; doi:10.1186/1471-2105-5-147)
Supplement: Additional File 2 — The original results of the above study (non-essential files are deleted to keep the file size under the limit set by BMC bioinformatics). [file 1471-2105-5-147-S2.bz2 › chilibotAdditionalFile2/dip20/342ID8409434E4302/html/IL7_IL7R.html]

 


 **IL7** and **IL7R** 
  
Found 103 abstracts in PubMed, retrieved 20.  
 

 What does Google say? 
 PDF only 
| .edu only 

---

**Interactive relationship** (e.g. stimulation, inhibition, etc)

**Neutral relationship**- The cellular basis underlying this dichotomy was revealed by the finding that the fetal IL 7R alpha  [ **IL7R** ]  population responded equally well to IL 7  [ **IL7** ]  and RANKL to express LT alpha beta.  Ref: 12479827 Immunity, 2002
- B lineage potential was largely associated with interleukin 7  [ **IL7** ]  receptor alpha IL 7R alpha  [ **IL7R** ]  expression and was selectively driven in culture by IL 7  [ **IL7** ] .  Ref: 12393656 Blood, 2002
- IL 7R alpha  [ **IL7R** ]  cells harvested from TRAF6 embryos expressed LTalphabeta in response to IL 7  [ **IL7** ]  but not RANKL, demonstrating that the RANK TRAF6 signaling pathway regulates LT alpha beta expression in LN but not in PP.  Ref: 12479827 Immunity, 2002
- Soluble IL 7  [ **IL7** ]  administered to TRAF6 embryos was sufficient to restore LN genesis indicating the functional similarities of the IL 7R alpha  [ **IL7R** ]  inducer cells for LN and PP genesis.  Ref: 12479827 Immunity, 2002

**Non-interactive relationship** (e.g. studied together, co-existance, homology, etc.)

- We have investigated the interleukin 7  [ **IL7** ]  receptor IL 7R alpha  [ **IL7R** ]  chain gene as a positional and functional candidate gene for susceptibility to multiple sclerosis MS, in view of its chromosomal location on 5p14 p12, a region that has shown suggestive linkage in MS genome screens, and its role in T and B cell proliferation and reactivity.  Ref: 12825072 Eur J Hum Genet, 2003
